# QTLs associated with agronomic traits in the Attila × CDC Go spring wheat population evaluated under conventional management

**DOI:** 10.1371/journal.pone.0171528

**Published:** 2017-02-03

**Authors:** Jun Zou, Kassa Semagn, Muhammad Iqbal, Hua Chen, Mohammad Asif, Amidou N’Diaye, Alireza Navabi, Enid Perez-Lara, Curtis Pozniak, Rong-Cai Yang, Harpinder Randhawa, Dean Spaner

**Affiliations:** 1 Department of Agricultural, Food and Nutritional Science, University of Alberta, Edmonton, Canada; 2 National Institute for Genomics and Advanced Biotechnology, National Agricultural Research Centre, Islamabad, Pakistan; 3 Department of Agronomy, 2004 Throckmorton Plant Science Center, Kansas State University, Manhattan, Kansas, United States of America; 4 Heartland Plant Innovations, Kansas Wheat Innovation Center, Manhattan, Kansas, United States of America; 5 Crop Development Centre and Department of Plant Sciences, University of Saskatchewan, Saskatoon, Canada; 6 Department of Plant Agriculture, University of Guelph, Guelph, Ontario, Canada; 7 Alberta Agriculture and Forestry, St. Edmonton, Alberta, Canada; 8 Agriculture and Agri-Food Canada, Lethbridge, Alberta, Canada; Institute of Genetics and Developmental Biology Chinese Academy of Sciences, CHINA

## Abstract

Recently, we investigated the effect of the wheat 90K single nucleotide polymorphic (SNP) array and three gene-specific (*Ppd-D1*, *Vrn-A1* and *Rht-B1*) markers on quantitative trait loci (QTL) detection in a recombinant inbred lines (RILs) population derived from a cross between two spring wheat (*Triticum aestivum* L.) cultivars, ‘Attila’ and ‘CDC Go’, and evaluated for eight agronomic traits at three environments under organic management. The objectives of the present study were to investigate the effect of conventional management on QTL detection in the same mapping population using the same set of markers as the organic management and compare the results with organic management. Here, we evaluated 167 RILs for number of tillers (tillering), flowering time, maturity, plant height, test weight (grain volume weight), 1000 kernel weight, grain yield, and grain protein content at seven conventionally managed environments from 2008 to 2014. Using inclusive composite interval mapping (ICIM) on phenotypic data averaged across seven environments and a subset of 1203 informative markers (1200 SNPs and 3 gene specific markers), we identified a total of 14 QTLs associated with flowering time (1), maturity (2), plant height (1), grain yield (1), test weight (2), kernel weight (4), tillering (1) and grain protein content (2). Each QTL individually explained from 6.1 to 18.4% of the phenotypic variance. Overall, the QTLs associated with each trait explained from 9.7 to 35.4% of the phenotypic and from 22.1 to 90.8% of the genetic variance. Three chromosomal regions on chromosomes 2D (61–66 cM), 4B (80–82 cM) and 5A (296–297 cM) harbored clusters of QTLs associated with two to three traits. The coincidental region on chromosome 5A harbored QTL clusters for both flowering and maturity time, and mapped about 2 cM proximal to the *Vrn-A1* gene, which was in high linkage disequilibrium (0.70 ≤ r^2^ ≤ 0.75) with SNP markers that mapped within the QTL confidence interval. Six of the 14 QTLs (one for flowering time and plant height each, and two for maturity and kernel weight each) were common between the conventional and organic management systems, which suggests issues in directly utilizing gene discovery results based on conventional management to make in detail selection (decision) for organic management.

## Background

More than 85% of wheat in Canada is produced in the western Canadian prairie provinces of Alberta, Saskatchewan and Manitoba, with a small proportion in British Columbia and eastern Canada [1]. Wheat breeders in western Canada primarily develop short stature cultivars that are early maturing, high yielding with high protein content and elevated dough strength. Currently, wheat cultivars to be registered in western Canada must have at least intermediate resistance to leaf rust (*Puccinia triticina Eriks*. *f*. *sp*. *tritici*), stripe rust (*P*. *striiformis* f. sp. *tritici*), stem rust (*P*. *graminis* f. sp. *tritici*), common bunt (caused both by *Tilletia tritici* and *Tilletia laevis*) and Fusarium head blight (caused mainly by *Fusarium graminearum*) (http://www.pgdc.ca). The availability of various improved wheat cultivars not only for good agronomic characteristics and quality traits, but also with better resistance to wheat diseases, have increased average wheat productivity in Canada by approximately four-fold from 0.8 Mg ha^-1^ in 1961 to 3.1 Mg ha^-1^ in 2014 (http://faostat3.fao.org). Wheat is grown in the region both under conventional and organic management systems, with the demand for organic production intensified in the last decade for different reasons, including concerns on human health, food quality and environment [2–4]. Conventional management system depends on high inorganic fertilizer and high pesticides and herbicides. On the contrary, organic management system relies on the use of (i) organic residues as soil amendments; (ii) biological nitrogen fixation, compost, manure and green manure as the major source of nutrients; (iii) mixed cropping, crop rotation and cover crops to minimize bare fallow; (iv) biological pest control; and (v) diverse plant species to minimize weeds and pests, support below-ground processes and to control soil erosion (www.intechopen.com). Most wheat breeders in western Canada develop semi-dwarf cultivars, which require high inputs (high nitrogen fertilizers, high pesticides and herbicides) to produce high grain yield and attain satisfactory protein content, but such cultivars types often produce lower grain yield in organic management due to weaker weed competitiveness [5]. Taller plants exhibit better competitive ability against weeds in organic management than shorter ones, mainly due to better light interception [6–8], but suffer lodging under high input demanding conventional management. Although most traits of interest in breeding for conventional management are similar to organic management, some of the traits relevant to the high-input demanding conventional farming may have negative effects in organic systems.

The Wheat Breeding group at the University of Alberta has been evaluating the performance of wheat cultivars and breeding lines under both conventional and organic management systems in Alberta, Canada [6, 7, 9–17]. In one of the recent studies [9], we evaluated a recombinant inbred line (RIL) population developed from a cross between ‘Attila’ (CM85836-50Y-0M-0Y-3M-0Y) [18] and ‘CDC Go’ in 2008, 2009 and 2010 under conventionally and organically managed field conditions and genotyped the population with 579 diversity arrays technology (DArT) and the *Rht-B1* gene specific markers. The soil at both organically and conventionally managed sites was an Udic Boroll (Orthic Black Chernozem in Canadian system) with loam or clay loam texture, neutral pH (6.7 to 7.4), and high soil organic matter (6 to 11%). The crop rotation on the conventionally managed site was wheat-pea-canola, while it was wheat followed by a green manure rye plow-down in the organic management site. The conventionally managed sites received 36–40 kg ha^-1^ fertilizer (11–52–0 N–P_2_O_5_–K_2_O) banded with seed at the time of planting, while the organically managed sites received neither chemical fertilizer nor compost [9]. Using the averaged phenotypic data across three environments, (i) we uncovered three QTLs under conventional management that were associated with plant height, grain yield and test weight, but none for tillering, kernel weight, grain protein content, days to flowering and maturity; (ii) we found five QTLs under organic management that were associated with plant height, test weight, grain protein content and kernel weight, but none for flowering time, maturity, number of tillers (tillering) and grain yield; and (iii) only a single QTL for plant height on 4B was common between the conventional and organic management systems. No QTL was identified for flowering time, maturity and tillering averaged across three environments both under conventional and organic management systems. Although several factors might have contributed to our failure to identify QTLs explaining most of the phenotypic variance in the ‘Attila’ × ‘CDC Go’ RIL population, low marker density and uneven marker distribution in the linkage maps are possible reasons. Currently, a total of 81,587 gene-associated SNPs (90K) is available for wheat genotyping through the Illumina iSelect SNP array [19], of which at least 5 to 13% could be polymorphic in a given bi-parental mapping population [20–23]. In order to investigate if an increase in marker density improves QTL detection, we reanalyzed the same phenotype data averaged over the three organically managed environments with a subset of 1200 high quality SNPs out of the 90K SNP array and three gene specific markers (*Ppd-D1*, *Vrn-A1* and *Rht-B1*) [20]. That study identified a total of 16 QTLs distributed across 10 chromosomes of which 13 QTLs were not reported using the DArT-based low-marker-density. The objectives of the present study were to (1) investigate if the 90K SNPs improve QTL detection in the ‘Attila’ and ‘CDC Go’ RIL population evaluated across seven environments under conventional management; and (2) compare the results with our previous studies conducted under organic management.

## Materials and methods

### Phenotyping and genotyping

The present study was conducted on a mapping population of 167 RILs developed from a cross between two spring wheat cultivars—‘Attila’ (CM85836-50Y-0M-0Y-3M-0Y) and ‘CDC Go’. As described in our previous studies [9, 20], ‘Attila’ is a semi dwarf, early maturing and medium yielding cultivar from the International Maize and Wheat Improvement Center [18], while ‘CDC Go’ is a medium height, relatively late maturing and high yielding Canadian western red spring wheat cultivar. The RIL population and the two parents were initially phenotyped under conventionally managed field conditions thrice from 2008 to 2010 at the Crop Research facility of the University of Alberta South Campus (53°19’N, 113°35’W), Edmonton, Canada [9]. Additional phenotypic data were obtained for four years (environments) by phenotyping the population from 2011 to 2014 at the same location. Each field experiment was conducted in a randomized incomplete block design with three replications in 2008 and 2009, and two replications in all other years. Details on crop rotation, input applications and all agronomic practices have been described in our previous study [9]. Each entry was evaluated for flowering and maturity time, number of tillers, plant height, test weight, thousand kernel weight, grain yield and grain protein content, as described in our previous study [9].

DNA extraction and genotyping was done as described in our previous studies [20, 21]. Briefly, DNA was extracted from three weeks old seedlings using a modified Cetyl Trimethyl Ammonium Bromide (CTAB) method. DNA samples were genotyped at the University of Saskatchewan Wheat Genomics lab, Saskatoon, Canada, with the Wheat 90K Illumina iSelect SNP array that consisted of 81,587 SNPs [19]. SNP alleles were called with the Illumina Genome Studio Polyploid Clustering version 1.0 software (Illumina, San Diego, USA) using default clustering parameters and filtered as described in our previous study [21]. In addition, the RILs and the two parents were also genotyped with *Ppd-D1* [24], *Vrn-A1* [25] and *Rht-B1* [26] gene specific markers at the Agricultural Genomics and Proteomics lab, University of Alberta, Edmonton, Canada as described elsewhere [21].

### Data analyses

Linkage analysis was performed as described in the ‘Cutler’ × ‘AC Barrie’ mapping population [21], while all other statistical analyses, including descriptive statistics, test for normality, F statistics, heritability and ICIM were conducted as described in one of our recent study [20]. Briefly, least squares means, variance statistics, and heritability were computed for each environment separately and then averaged across all environments using PROC MIXED and PROC IML in SAS version 9.3 (SAS Institute Inc. Cary, USA). Genotypes (RILs) were considered fixed, while years, replications and blocks within replications were considered as random effects. Both test for normality and the frequency distribution were computed using MiniTab v14. ICIM was performed on the least squares means of each trait for individual environment and averaged across all environments with QTL IciMapping v4.0 [27, 28] using a mean replacement for missing data, 1 cM walking distance, a minimum logarithm of odds (LOD) score of 2.5 and a model to determine additive effects at individual QTL and additive × additive epistatic interactions. QTL names were designated following the International Rules of Genetic Nomenclature (http://wheat.pw.usda.gov/ggpages/wgc/98/Intro.htm), which consisted of three letters for trait acronym, lab designation (dms = Dean Michael Spaner) and chromosome. Genetic maps and QTL graphs were drawn using MapChart v2.1 [29]. The extent of linkage disequilibrium (LD) between the *Ppd-D1*, *Rht-B1* and *Vrn-A1* gene specific markers and all SNPs that mapped on chromosomes 2D, 4B and 5A, respectively, was evaluated by computing the *r*^2^ values using TASSEL version 5.2.30 [30].

## Results

### Summary of phenotypic traits and markers

Table 1 provides a summary of the descriptive statistics of the two parents and RILs plus F statistics of the 167 RILs evaluated under conventional management across seven (2008–2014) years (environments). ‘CDC Go’ matured about 3 days earlier, produced 57 more tillers m^-2^, with kernels 2 g heavier and 0.6% higher grain protein content, but was 2 cm taller and yielded 277 kg ha^-1^ less grain than ‘Attila’. In our previous study conducted from 2008 to 2010, ‘CDC Go’ yielded approximately 420 kg ha^-1^ more grain than ‘Attila’ [9], but it yielded lower grain when the data were averaged across all seven environments (Table 1). The 167 RILs varied in height from 63 to 102 cm, required 48–60 days to flowering and 93–105 days to maturity, and yielded from 3.5 to 5.9 Mg ha^-1^ grain. The phenotypic distribution of least square means averaged across seven environments was normal (P > 0.050) for all traits, except test weight (S1 Fig). The Shapiro-Wilk test rejected the hypothesis of normality (P = 0.018) for test weight. Averaged across all seven environments, genotypes differed (p < 0.001) for all traits (Table 1). Broad sense heritability varied from 0.25 for number of tillers to 0.73 for flowering time (Table 2).

**Table 1:** Summary of least squares means and F statistics of 167 recombinant inbred lines (RILs) derived from ‘Attila’ × ‘CDC Go’ and evaluated across seven (2008–2014) conventionally managed environments in Edmonton, Canada.

	Parents		RILs (descriptive and F statistics)					
Trait	‘Attila’	‘CDC Go’	Min	Max	Mean	SD	CV (%)	F value*
No. of tillers (m^-2^)	419.6	476.9	393.8	558.6	475.1	6.5	6.1	2.7
Flowering time (days)	54.4	50.6	48.4	60.1	53.3	2.5	4.7	20.7
Maturity time (days)	99.3	96.1	92.8	105.1	97.9	2.7	2.7	5.0
Plant height (cm)	75.1	77.1	62.5	102.0	81.5	8.3	10.2	24.4
Grain yield (Mg ha^-1^)	5.4	5.1	3.5	5.9	4.7	5.4	11.4	10.8
Test weight (kg hL^-1^)	77.6	77.7	74.7	79.1	77.0	0.8	1.1	3.6
1000-kernels weight (g)	38.2	39.9	34.8	42.6	39.0	1.7	4.4	2.6
Grain protein content (%)	12.3	12.9	10.9	13.7	12.4	0.6	4.5	4.3

* All F-values were significant at p < 0.001.

**Table 2:** Comparisons of QTLs associated with eight agronomic traits in our previous study [9] and present study. The previous study was based on averaged phenotypic data of three (2008–2010) conventionally managed environments and genotypic data of 579 DArT and the *Rht-B1* gene specific markers, while the present study was based seven environments (2008–2014) and 1203 SNP and gene specific markers.

Trait	Heritability		Number of QTLs identified		Total phenotypic variance explained by all QTLs (%)		Genetic variance (%) explained by all QTLs		Difference between the two studies: Present minus previous (%)*	
	Previous	Present	Previous	Present	Previous	Present	Previous	Present	Phenotypic variance	Genetic variance
Flowering time	0.76	0.73	0	1	0.0	16.8	0.0	23.0	16.8	23.0
Maturity	0.38	0.45	0	2	0.0	29.9	0.0	66.5	29.9	66.5
Plant height	0.58	0.62	1	1	19.2	18.4	33.1	29.7	-0.8	-3.5
Thousand kernels weight	0.37	0.39	0	4	0.0	35.4	0.0	90.8	35.4	90.8
Test weight	0.28	0.35	1	2	10.9	16.2	38.9	46.4	5.3	7.4
Grain yield	0.37	0.44	1	1	17.0	9.3	45.9	21.1	-7.7	-24.8
Number of tillers	0.32	0.25	0	1	0.0	11.2	0.0	44.8	11.2	44.8
Grain protein content	0.64	0.26	0	2	0.0	18.6	0.0	71.7	18.6	71.7

* Differences in phenotypic and genetic variance explained in the present study using SNPs minus the previous study using DArT markers. For both plant height and grain yield, more variation was explained in the previous study than the current study, which resulted to negative values.

Detailed results on the SNP and gene specific markers and linkage maps used in the present study were presented in our previous study [20]. After excluding co-segregating SNPs, a subset of 1203 markers (1200 SNPs plus *Ppd-D1*, *Vrn-A1a*, and *Rht-B1*) were used for QTL mapping. The number of markers retained for QTL mapping varied from 4 on chromosome 1D to 150 on 2B (S1 Table). There were no polymorphic markers for both chromosomes 3D and 4D. The total map length for the 19 chromosomes (excluding chromosome 3D and 4D) was 3442 cM, with each chromosome varying from 14.3 cM on 1D to 324.8 cM on 5B. Map distance between adjacent markers varied from 0.6 to 48.8 cM, and the overall average was 2.9 cM [20].

### QTLs under conventional management

**Fig 1:**
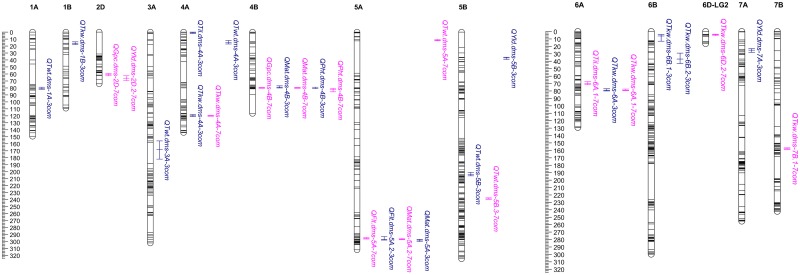
The distribution of QTLs associated with eight agronomic traits evaluated across three (2008–2010) organically managed environments (blue font) and seven (2008–2014) conventionally managed environments (pink font). Map position in centiMorgans (cM) is shown on the left side of the chromosomes, with each horizontal line representing a marker. QTLs are shown on the right side of each linkage group, with bars indicating their 95% genetic confidence interval. Details of each QTL under conventional management is given in Table 3, while those QTLs under organic management is given in S2 Table.

The analyses conducted using the averaged least squares means phenotypic data of the seven environments uncovered a total of 14 QTLs (Fig 1 and Table 3), which included one QTL each for tillering per m^2^ (*QTil*.*dms-6A*.*1*), flowering time (*QFlt*.*dms-5A*), plant height (*QPht*.*dms-4B*) and grain yield (*QYld*.*dms-2D*.*2*); two QTLs each for maturity (*QMat*.*dms-4B* and *QMat*.*dms-5A*.*2*), grain protein content (*QGpc*.*dms-2D*.*2* and *QGpc*.*dms-4B*) and test weight (*QTwt*.*dms-5A* and *QTwt*.*dms-5B*.*3*); and four QTLs for kernel weight (*QTkw*.*dms-4A*, *QTkw*.*dms-6A*.*1*, *QTkw*.*dms-6D*.*2* and *QTkw*.*dms-7B*.*1*). All QTLs associated with each trait exhibited mainly additive effects and QTL by QTL interactions were negligible (R^2^ < 2%). The QTL for tillering mapped at 70 cM on chromosome 6A (*QTil*.*dms-6A*.*1*) and accounted for 11.2% of the phenotypic variance across seven environments. RILs that had ‘Attila’ alleles at the two flanking markers for *QTil*.*dms-6A*.*1*, on average, had 3.4 more tillers than those RILs homozygous for ‘CDC Go’ alleles. However, this QTL was not detected in any of the individual environments; instead, we found 6 other environment specific QTLs on 4A (*QTil*.*dms-4A*), 5A (*QTil*.*dms-5A*), 6A (*QTil*.*dms-6A*.*2)* and 7A (*QTil*.*dms-7A*.*1*, *QTil*.*dms-7A*.*2* and *QTil*.*dms-7A*.*3*) that were associated with tillering in 2009, 2013 and/or 2014 environments (Table 3).

**Table 3:** Summary of QTLs associated with the different traits based on 167 recombinant inbred line (RIL) population evaluated at seven environments from 2008 to 2014 under conventional management system. QTL analyses were conducted using least squares means of each environment and averaged (combined) across all seven environments.

QTL	Trait*	Environment	Chrom.	Position (cM)	Confidence interval (cM)	Left Marker	Right Marker	LOD	R^2^ (%)	Additive effect	Difference**
*QFlt*.*dms-4A*	Fllt	2014	4A	1	0–3.5	Excalibur_c82040_91	wsnp_Ra_rep_c70233_67968353	3.0	8.5	-0.6	
*QFlt*.*dms-4B*	Fllt	2010	4B	101	90.5–113.5	wsnp_Ra_c1146_2307483	Rht-B1	2.5	9.0	0.9	
*QFlt*.*dms-4B*	Fllt	2011	4B	101	90.5–113.5	wsnp_Ra_c1146_2307483	Rht-B1	2.5	9.0	0.9	
*QFlt*.*dms-5A*	Fllt	2009	5A	289	285.5–290.5	Ra_c3966_2205	Tdurum_contig10843_745	5.2	13.0	-1.0	
*QFlt*.*dms-5A*	Fllt	2014	5A	293	290.5–293.5	wsnp_Ex_rep_c101994_87256479	Excalibur_c26671_282	4.4	12.6	-0.8	
*QFlt*.*dms-5A*	Fllt	2010	5A	296	294.5–296.5	Vrn-A1	Kukri_c12384_430	6.8	13.0	-1.1	
*QFlt*.*dms-5A*	Fllt	2011	5A	296	294.5–296.5	Vrn-A1	Kukri_c12384_430	6.8	13.0	-1.1	
*QFlt*.*dms-5A*	Fllt	Combined	5A	296	294.5–296.5	Vrn-A1	Kukri_c12384_430	6.6	16.8	-1.0	-2.5
*QFlt*.*dms-6B*	Fllt	2009	6B	232	230.5–232.5	wsnp_Ex_c18632_27501906	Tdurum_contig32579_121	3.3	7.6	0.8	
*QGpc*.*dms-2B*	Gpc	2013	2B	1	0–4.5	IAAV7130	wsnp_BG274584B_Ta_2_1	2.5	6.8	0.2	
*QGpc*.*dms-2D*	Gpc	2009	2D	61	58.5–62.5	BS00011109_51	Excalibur_c24307_739	4.4	8.9	-0.3	
*QGpc*.*dms-2D*	Gpc	Combined	2D	61	59.5–62.5	BS00011109_51	Excalibur_c24307_739	9.1	13.4	0.2	0.3
*QGpc*.*dms-2D*	Gpc	2010	2D	65	62.5–68.5	RAC875_rep_c73531_335	wsnp_Ex_c8303_14001708	6.0	12.0	-0.4	-375.7
*QGpc*.*dms-3A*	Gpc	2008	3A	86	84.5–88.5	RAC875_c6006_105	Tdurum_contig34075_98	2.7	7.2	-0.2	-422.2
*QGpc*.*dms-4B*	Gpc	2010	4B	79	77.5–79.5	RAC875_c47018_72	Tdurum_contig29054_113	5.4	10.8	-0.4	
*QGpc*.*dms-4B*	Gpc	Combined	4B	80	79.5–80.5	Tdurum_contig29054_113	wsnp_Ra_c3790_6990678	3.4	6.3	-0.2	-0.5
*QGpc*.*dms-5B*	Gpc	2009	5B	15	1.5–26.5	BS00062618_51	wsnp_Ex_c3874_7036132	3.1	8.3	0.3	
*QYld*.*dms-2D*.*1*	Yld	2009	2D	4	0–17.5	Ppd-D1	GENE-0787_85	2.7	8.4	-178.2	
*QYld*.*dms-2D*.*1*	Yld	2011	2D	9	0–19.5	Ppd-D1	GENE-0787_85	3.9	11.7	-313.1	
*QYld*.*dms-2D*.*2*	Yld	2014	2D	65	62.5–69.5	RAC875_rep_c73531_335	wsnp_Ex_c8303_14001708	3.7	9.2	197.7	
*QYld*.*dms-2D*.*2*	Yld	2013	2D	66	62.5–70.5	RAC875_rep_c73531_335	wsnp_Ex_c8303_14001708	4.1	10.5	212.3	
*QYld*.*dms-2D*.*2*	Yld	Combined	2D	66	62.5–69.5	RAC875_rep_c73531_335	wsnp_Ex_c8303_14001708	2.9	9.3	-153.3	-375.7
*QYld*.*dms-2D*.*2*	Yld	2010	2D	67	62.5–69.5	RAC875_rep_c73531_335	wsnp_Ex_c8303_14001708	4.2	11.1	253.0	
*QYld*.*dms-2D*.*2*	Yld	2011	2D	68	63.5–70.5	RAC875_rep_c73531_335	wsnp_Ex_c8303_14001708	3.7	6.0	224.9	
*QYld*.*dms-3A*.*1*	Yld	2008	3A	9	3.5–12.5	wsnp_Ex_c15475_23757972	IACX6065	2.9	7.4	260.6	
*QYld*.*dms-3A*.*2*	Yld	2014	3A	116	112.5–116.5	BS00048633_51	RAC875_rep_c109228_400	4.3	10.3	202.8	
*QYld*.*dms-6B*	Yld	2009	6B	229	228.5–230.5	BS00067388_51	Excalibur_c7713_272	4.1	10.9	197.8	
*QYld*.*dms-7A*	Yld	2013	7A	48	46.5–55.5	Kukri_c18440_92	Kukri_rep_c75743_357	3.2	7.5	-179.3	
*QYld*.*dms-7A*	Yld	2011	7A	56	50.5–58.5	Kukri_c18440_92	Kukri_rep_c75743_357	2.8	6.3	-194.0	
*QYld*.*dms-7D*	Yld	2013	7D	38	34.5–41.5	Excalibur_c4508_1959	wsnp_Ex_c17914_26681837	3.3	7.7	181.9	
*QYld*.*dms-7D*	Yld	2014	7D	38	37.5–45.5	Excalibur_c4508_1959	wsnp_Ex_c17914_26681837	3.4	7.7	176.6	
*QMat*.*dms-2D*.*1*	Mat	2009	2D	0	0–14.5	Ppd-D1	GENE-0787_85	2.5	0.8	-0.5	
*QMat*.*dms-2D*.*2*	Mat	2013	2D	68	63.5–74	RAC875_rep_c73531_335	wsnp_Ex_c8303_14001708	2.8	4.7	0.9	
*QMat*.*dms-4B*	Mat	2009	4B	72	68.5–75.5	tplb0026o15_1634	BobWhite_c4311_148	3.4	7.6	0.7	
*QMat*.*dms-4B*	Mat	2010	4B	78	77.5–78.5	wsnp_Ex_c35910_43971560	RAC875_c47018_72	9.4	19.7	2.2	
*QMat*.*dms-4B*	Mat	2013	4B	80	79.5–80.5	Tdurum_contig29054_113	wsnp_Ra_c3790_6990678	9.7	17.7	1.8	
*QMat*.*dms-4B*	Mat	2014	4B	80	78.5–80.5	Tdurum_contig29054_113	wsnp_Ra_c3790_6990678	5.1	11.4	0.8	
*QMat*.*dms-4B*	Mat	Combined	4B	80	79.5–80.5	Tdurum_contig29054_113	wsnp_Ra_c3790_6990678	7.6	15.9	1.1	2.2
*QMat*.*dms-4B*	Mat	2012	4B	81	80.5–81.5	RAC875_c103110_275	RAC875_c24550_1150	3.7	7.4	1.0	
*QMat*.*dms-5A*.*2*	Mat	2009	5A	292	290.5–293.5	Tdurum_contig10843_745	wsnp_Ex_rep_c101994_87256479	4.7	9.7	-0.8	
*QMat*.*dms-5A*.*2*	Mat	Combined	5A	297	295.5–297.5	Kukri_c12384_430	wsnp_Ex_c22727_31934296	6.8	14.0	-1.0	-1.9
*QMat*.*dms-5A*.*2*	Mat	2010	5A	298	296.5–298.5	wsnp_Ex_c22727_31934296	wsnp_Ex_rep_c66689_65010988	3.3	6.3	-1.2	
*QMat*.*dms-5A*.*2*	Mat	2014	5A	301	300.5–301.5	wsnp_Ex_c2702_5013188	Excalibur_rep_c111129_125	5.6	12.7	-0.9	
*QMat*.*dms-5A*.*2*	Mat	2013	5A	310	304.5–311	BS00044408_51	wsnp_Ex_c27046_36265198	3.5	6.2	-1.0	
*QMat*.*dms-5B*	Mat	2010	5B	56	54.5–57.5	BS00048316_51	IAAV8455	2.6	5.0	-1.1	
*QMat*.*dms-6B*.*1*	Mat	2012	6B	24	19.5–26.5	Ex_c20409_854	wsnp_Ex_c19082_27999258	6.0	13.3	1.3	
*QMat*.*dms-6B*.*2*	Mat	2012	6B	130	128.5–130.5	wsnp_BM134512B_Ta_2_1	wsnp_Ra_c14498_22667649	2.9	5.8	-0.8	
*QMat*.*dms-7A*	Mat	2013	7A	61	60.5–61.5	wsnp_Ra_rep_c105976_89839782	BobWhite_c911_127	3.4	5.7	-1.0	
*QPht*.*dms-2D*.*2*	Pht	2009	2D_LG2	1	0–1.5	wsnp_CAP7_rep_c5643_2537213	D_F5XZDLF02HWOJZ_227	3.1	8.5	2.1	
*QPht*.*dms-2D*.*2*	Pht	2010	2D_LG2	1	0–1.5	wsnp_CAP7_rep_c5643_2537213	D_F5XZDLF02HWOJZ_227	2.7	3.2	2.2	
*QPht*.*dms-4B*	Pht	2013	4B	81	80.5–84.5	RAC875_c103110_275	RAC875_c24550_1150	8.2	20.0	-5.2	
*QPht*.*dms-4B*	Pht	2010	4B	82	80.5–84.5	RAC875_c24550_1150	wsnp_Ra_c1146_2307483	8.5	11.2	-4.2	
*QPht*.*dms-4B*	Pht	2011	4B	82	80.5–86.5	RAC875_c24550_1150	wsnp_Ra_c1146_2307483	5.2	13.4	-3.4	
*QPht*.*dms-4B*	Pht	2012	4B	82	80.5–84.5	RAC875_c24550_1150	wsnp_Ra_c1146_2307483	9.0	22.6	-4.5	
*QPht*.*dms-4B*	Pht	2014	4B	82	80.5–84.5	RAC875_c24550_1150	wsnp_Ra_c1146_2307483	10.3	23.9	-5.0	
*QPht*.*dms-4B*	Pht	Combined	4B	82	80.5–85.5	RAC875_c24550_1150	wsnp_Ra_c1146_2307483	7.3	18.4	-3.8	-7.7
*QPht*.*dms-5A*	Pht	2013	5A	292	290.5–293.5	Tdurum_contig10843_745	wsnp_Ex_rep_c101994_87256479	3.7	8.4	-3.3	
*QPht*.*dms-6B*.*1*	Pht	2010	6B	113	112.5–118.5	wsnp_Ex_c3101_5719964	wsnp_Ra_rep_c73731_71807419	5.3	6.5	-3.0	
*QPht*.*dms-6B*.*2*	Pht	2010	6B	160	158.5–161.5	Kukri_c59960_211	Ku_c59960_1939	10.2	13.3	4.4	
*QTil*.*dms-4A*	Til	2009	4A	0	0–1.5	Excalibur_c82040_91	wsnp_Ra_rep_c70233_67968353	3.3	10.2	3.9	
*QTil*.*dms-5A*	Til	2013	5A	223	221.5–237.5	Excalibur_c47920_249	RAC875_c2061_292	4.8	6.4	6.3	
*QTil*.*dms-6A*.*1*	Til	Combined	6A	70	67.5–71.5	wsnp_BG262421A_Ta_2_2	wsnp_Ku_c38451_47086066	3.3	11.2	-2.0	-3.4
*QTil*.*dms-6A*.*2*	Til	2013	6A	84	83.5–86.5	BS00066623_51	BobWhite_c10342_117	3.0	3.8	-5.0	
*QTil*.*dms-7A*.*1*	Til	2013	7A	8	1.5–12.5	wsnp_Ra_rep_c69620_67130107	GENE-5000_606	3.9	5.9	-6.1	
*QTil*.*dms-7A*.*2*	Til	2013	7A	77	71.5–87.5	RAC875_c29361_70	Excalibur_c49272_174	6.8	12.9	9.2	
*QTil*.*dms-7A*.*3*	Til	2014	7A	214	206.5–219.5	BS00068575_51	wsnp_Ku_c28104_38042857	3.7	9.8	-4.0	
*QTkw*.*dms-2B*	Tkw	2008	2B	288	284.5–289.5	Kukri_c15782_491	Tdurum_contig54649_798	2.6	6.5	0.8	
*QTkw*.*dms-3A*.*1*	Tkw	2009	3A	214	213.5–214.5	wsnp_Ex_c16864_25440739	RAC875_rep_c113506_409	2.8	3.0	-0.6	
*QTkw*.*dms-4A*	Tkw	2009	4A	120	118.5–120.5	wsnp_Ex_c7899_13416443	wsnp_Ex_rep_c97236_84366722	9.3	10.7	1.2	
*QTkw*.*dms-4A*	Tkw	Combined	4A	120	119.5–120.5	wsnp_Ex_c7899_13416443	wsnp_Ex_rep_c97236_84366722	5.8	12.1	0.6	1.1
*QTkw*.*dms-5B*	Tkw	2008	5B	303	300.5–304.5	Kukri_rep_c106832_790	wsnp_JD_c38123_27754848	4.1	10.5	-1.0	
*QTkw*.*dms-6A*.*1*	Tkw	2009	6A	79	77.5–79.5	wsnp_Ku_rep_c112734_95776957	BS00036878_51	15.2	19.3	1.6	
*QTkw*.*dms-6A*.*1*	Tkw	Combined	6A	79	77.5–79.5	TA005366-0788	wsnp_Ku_rep_c112734_95776957	4.0	8.5	0.5	1.1
*QTkw*.*dms-6A*.*2*	Tkw	2009	6A	108	107.5–110.5	Kukri_c669_259	Excalibur_rep_c104696_400	6.4	7.0	-1.0	
*QTkw*.*dms-6D*.*2*	Tkw	Combined	6D_LG2	4	3.5–5.5	wsnp_Ex_c14691_22765150	wsnp_Ex_c14691_22763609	3.2	6.7	0.4	0.9
*QTkw*.*dms-7B*.*1*	Tkw	Combined	7B	158	156.5–158.5	wsnp_Ku_c17161_26193994	BS00003350_51	4.0	8.1	0.5	0.9
*QTwt*.*dms-2B*	Twt	2012	2B	109	108.5–110.5	RAC875_c85927_269	Kukri_c6973_344	2.6	7.0	-0.5	
*QTwt*.*dms-5A*	Twt	Combined	5A	12	10.5–12.5	wsnp_Ex_rep_c107017_90850230	RAC875_c9617_395	2.5	6.1	0.2	0.5
*QTwt*.*dms-5B*.*2*	Twt	2010	5B	163	160.5–163.5	Tdurum_contig53072_1935	Kukri_rep_c69276_59	3.7	9.9	-0.4	
*QTwt*.*dms-5B*.*3*	Twt	Combined	5B	239	237.5–239.5	wsnp_Ra_c26091_35652620	RAC875_c12552_233	4.4	10.1	-0.3	-0.5

* Flt: flowering time (days); Mat: maturity (days); Til: number of tillers or tillering ability (m^-2^); Pht: plant height (cm); Tkw: thousand kernels weight (g); Twt: test weight (kg hL^-1^); Yld: grain yield (kg ha^-1^); Gpc: grain protein content (%).

** Difference in least squares means of all RILs that had the ‘CDC Go’ alleles at both flanking markers of every QTL to those that had the ‘Attila’ alleles; it was calculated only for the averaged phenotypic data across seven environments. Positive and negative additive effects or phenotypic differences for grain yield, grain protein content, test weight, kernel weight and tillering ability indicate that the favorable alleles originated from ‘CDC Go’ and ‘Attila’, respectively; for flowering, maturity and plant height, positive and negative values indicate the opposite (the favorable alleles originated from ‘Attila’ and ‘CDC Go’, respectively), because selection is made against higher values (late flowering, late maturity and taller plants).

The QTL for flowering time mapped at 296 cM on chromosome 5A (*QFlt*.*dms-5A*), flanked by the *Vrn-A1* gene, and explained 16.8% of the phenotypic variance across the seven environments (Table 3). RILs with ‘CDC Go’ alleles at the two flanking markers for *QFlt*.*dms-5A* flowered 2.5 days earlier than those RILs homozygous for ‘Attila’ alleles. When individual environments were considered, *QFlt*.*dms-5A* was detected at the same confidence interval in four environments (2009, 2010, 2011 and 2014), and explained from 12.6 to 13.0% of phenotypic variance of the individual environments (Table 3). In addition, three environment specific QTLs for flowering time were also detected on 4A (*QFlt*.*dms-4A*), 4B (*QFlt*.*dms-4B*) and 6B (*QFlt*.*dms-6B*), which individually explained from 7.6 to 9.0% of the phenotypic variance. The two QTLs for maturity mapped at 80 cM on chromosome 4B (*QMat*.*dms-4B*) and at 297 cM on 5A (*QMat*.*dms-5A*.*2*), which individually explained 15.9 and 14.0%, respectively, and altogether accounted for 29.9% of the phenotypic variance across the seven environments. The favorable alleles for *QMat*.*dms-4B* and *QMat*.*dms-5A*.*2* originated from ‘Attila’ and ‘CDC Go’, respectively. RILs that were homozygous for the favorable alleles at the two flanking markers of each QTL matured about two days earlier than those RILs that were homozygous for the unfavorable alleles. When individual environments were considered, *QMat*.*dms-4B* and *QMat*.*dms-5A*.*2* were detected at the same confidence interval in five (2009 to 2014 except 2011) and four (2009, 2010, 2013 and 2014) out of the seven environments, respectively (Table 3). The proportion of phenotypic variance explained by *QMat*.*dms-4B* and *QMat*.*dms-5A*.*2* in individual environments varied from 7.4 to 19.4% and from 6.2 to 12.7%, respectively. Furthermore, we also found six environment specific QTLs for maturity on chromosomes 2D, 5B, 6B and 7A that individually explained from 0.8 to 13.3% of the phenotypic variance of the individual environments (Table 3).

The QTL associated with plant height across seven environments mapped at 82 cM on chromosome 4B (*QPht*.*dms-4B*) and explained 18.4% of the phenotypic variance. *Rht-B1* gene mapped 34.5 cM distal to *QPht*.*dms-4B* and 27 cM distal to one of the flanking SNP markers, wsnp_Ra_c1146_2307483. RILs that had the ‘CDC Go’ alleles at the two flanking markers for *QPht*.*dms-4B* were 7.7 cm shorter than those RILs that were homozygous for ‘Attila’ alleles (Table 3). When individual environments were considered, *QPht*.*dms-4B* was consistently detected at the same position in five (2010 to 2014) of the seven environments, but the proportion of phenotypic variance explained by *QPht*.*dms-4B* was variable, ranging from 11.9 to 23.9% (Table 3). We also found four environment specific QTLs for plant height on chromosomes 2D (*QPht*.*dms-2D*.*2*), 5A (*QPht*.*dms-5A*) and 6B (*QPht*.*dms-6B*.*1* and *QPht*.*dms-6B*.*2*), which individually explained from 3.2 to 13.3% of the phenotypic variance in the 2009, 2010 and 2013 environments, respectively (Table 3). We found one QTL for grain yield at 66 cM on 2D (*QYld*.*dms-2D*.*2*) that explained 9.3% of the phenotypic variance across the seven environments. The photoperiod response *Ppd-D1* gene mapped 66 cM distal to *QYld*.*dms-2D*.*2*. RILs with ‘Attila’ alleles at the two flanking markers for *QYld*.*dms-2D*.*2* produced 375.7 kg ha^-1^ more grain yield than those RILs homozygous for ‘CDC Go’ alleles. When individual environments were considered, *QYld*.*dms-2D*.*2* was detected at the same confidence interval in four (2010, 2011, 2013 and 2014) of the seven environments, explaining from 6.0 to 11.1% of the phenotypic variance at individual environments. In addition, five environment-specific QTLs associated with grain yield were also identified on chromosomes 3A, 6B, 7A and 7D explaining from 6.3 to 10.9% of the phenotypic variance (Table 3).

The two QTLs associated with test weight across the seven environments were located at 12 cM on chromosome 5A (*QTwt*.*dms-5A*) and at 239 cM on 5B (*QTwt*.*dms-5B*.*3*), and they explained 6.1 and 10.1% of the phenotypic variance, respectively (Table 3). The favorable alleles for *QTwt*.*dms-5A* and *QTwt*.*dms-5B*.*3* originated from ‘CDC Go’ and ‘Attila’, respectively. RILs homozygous for the favorable alleles at the two flanking markers for *QTwt*.*dms-5A* and *QTwt*.*dms-5B*.*3* had 0.5 kg hL^-1^ higher test weight than those RILs with unfavorable alleles. Neither *QTwt*.*dms-5A* nor *QTwt*.*dms-5B*.*3* were detected in any of the individual environments; instead, we found two environment specific QTLs at 109 cM on 2B and at 163 cM on 5B that individually explained 7.0 and 9.9%, respectively, of the phenotypic variance for test weight at individual environments (Table 3).

For kernel weight, we found four QTLs at 120 cM on 4A (*QTkw*.*dms-4A*), at 79 cM on 6A (*QTkw*.*dms-6A*.*1*), at 4 cM on 6D *(QTkw*.*dms-6D*.*2)* and at 158 cM on 7B *(QTkw*.*dms-7B*.*1*). Each QTL individually explained from 6.7 to 12.1% and altogether accounted for 35.4% of the phenotypic variance across seven environments (Table 3). RILs homozygous for ‘CDC Go’ alleles at the two flanking markers of each QTL were from 0.9 to 1.1 mg heavier per kernel than those with ‘Attila’ alleles. When individual environments were considered, both *QTkw*.*dms-4A* and *QTkw*.*dms-6A*.*1* were detected in the 2009 environment; all other QTLs were not detected in any of the individual environments. We also found four additional environment specific QTLs associated with kernel weight on 2B, 3A, 5B and 6A, explaining from 3.0 to 10.5% of the phenotypic variance (Table 3).

The two QTLs associated with grain protein content across seven environments mapped at 62 cM on 2D (*QGpc*.*dms-2D*) and at 80 cM on 4B (*QGpc*.*dms-4B*). *QGpc*.*dms-2D* and *QGpc*.*dms-4B* explained 13.4 and 6.3%, respectively, and together accounted for 19.7% of the phenotypic variance across seven environments (Table 3). The favorable alleles for *QGpc*.*dms-2D* and *QGpc*.*dms-4B* originated from ‘CDC Go’ and ‘Attila’, respectively. RILs homozygous for the favorable alleles at the two flanking markers of each QTL showed 0.5% higher grain protein content than those RILs homozygous for unfavorable alleles. When individual environments were considered, *QGpc*.*dms-2D* was detected in two (2009 and 2010) environments, while *QGpc*.*dms-4B* was detected only in 2010. In addition, three environment-specific QTLs on chromosomes 2B, 3A and 5B were detected that individually explained from 6.8 to 8.3% of the phenotypic variance at a single environment (Table 3).

### Chromosomal regions harbouring QTL clusters

The first coincidental QTL mapped on chromosome 5A and was associated with both flowering time (*QFlt*.*dms-5A*) and maturity (*QMat*.*dms-5A*.*2*) in the combined data across seven environments plus plant height (*QPht*.*dms-5A*) in 2013 environment (Fig 1, Table 3). This coincidental QTL explained from 14.0 to 16.8% of the phenotypic variance for flowering time and maturity across seven environments and from 8.4 to 14.6% of the phenotypic variance for plant height in two environments (Table 3). RILs carrying the ‘CDC Go’ alleles at the two flanking markers of the QTL on 5A were different (p ≤ 0.03) from those possessing ‘Attila’ alleles for flowering time, maturity, plant height and test weight, but not for the other four traits (Table 4). On average, RILs with the ‘CDC Go’ allele at the two flanking markers of the coincidental QTL on 5A flowered/matured 2 days earlier, were 4 cm shorter and had 0.3 kg hL^-1^ higher test weight than those RILs with ‘Attila’ alleles. The second coincidental QTL mapped on 4B (Fig 1, Table 3) and was associated with maturity (*QMat*.*dms-4B*), plant height (*QPht*.*dms-4B*) and grain protein content (*QGpc*.*dms-4B*). RILs with ‘Attila’ alleles at the two flanking markers for this QTL on 4B were different (p ≤ 0.008) than those with ‘CDC Go’ alleles for maturity, plant height and grain protein content, but not for the other five traits (Table 4). RILs carrying the ‘Attila’ alleles at the two flanking markers matured 2.2 days earlier and had 0.3% higher grain protein, but were 7.6 cm taller than those homozygous for ‘CDC Go’ alleles (Table 4). The third coincidental QTL mapped on 2D (Fig 1, Table 3) and was associated with both grain yield (*QYld*.*dms-2D*) and grain protein content (*QGpc*.*dms-2D*). RILs carrying ‘CDC Go’ alleles at the two flanking markers for the coincidental QTL on 2D were different (p ≤ 0.001) from those possessing ‘Attila’ alleles for both grain yield and grain protein content, but not for the other six traits (Table 4). RILs carrying ‘Attila’ alleles at the two flanking markers yielded 335.9 kg ha^-1^ more grain with 0.4% lower grain protein content than those homozygous for ‘CDC Go’ alleles (Table 4).

**Table 4:** Comparisons of recombinant inbred lines that had the ‘CDC Go’ or ‘Attila’ alleles at the flanking markers of three coincident QTLs on eight traits evaluated under seven (2008–2014) conventional management environments. RILs with recombinant genotypes at the flanking markers of each coincident QTL were excluded from analysis.

Trait	Chromosome	Coincident QTL name	‘Attila’ type alleles	‘CDC Go’ type alleles	Difference*	F statistics	p value
Flowering time (days)	2D	*QGpc*.*dms-2D vs QYld*.*dms-2D*.*2*	53.90	52.90	-1.00	3.00	0.087
Maturity (days)	2D		98.40	97.70	-0.70	1.00	0.317
Number of tillers (m^-2^)	2D		107.00	106.90	-0.10	0.01	0.938
Plant height (cm)	2D		82.90	80.70	-2.20	1.50	0.217
1000 kernel weight (g)	2D		38.90	39.20	0.30	0.81	0.369
Test weight (kg hL^-1^)	2D		77.00	77.00	0.00	0.03	0.869
Grain yield (Mg ha^-1^)	2D		4.95	4.61	-0.34	16.00	0.001
Grain protein content (%)	2D		12.10	12.50	0.40	23.10	0.001
Flowering time (days)	4B	*QGpc*.*dms-4B vs QMat*.*dms-4B vs QPht*.*dms-4B*	53.20	53.80	0.60	1.00	0.324
Maturity (days)	4B		97.10	99.20	2.10	25.00	0.001
Number of tillers (m^-2^)	4B		106.60	107.80	1.20	0.50	0.473
Plant height (cm)	4B		83.90	76.30	-7.60	30.10	0.001
1000 kernel weight (g)	4B		39.10	38.70	-0.40	1.90	0.240
Test weight (kg hL^-1^)	4B		77.10	77.00	-0.10	1.20	0.280
Grain yield (kg ha^-1^)	4B		4.74	4.78	0.04	0.40	0.538
Grain protein content (%)	4B		12.50	12.25	-0.25	7.10	0.008
Flowering time (days)	5A	*QFlt*.*dms-5A vs QMat*.*dms-5A*.*2 vs QPht*.*dms-5A*	54.50	52.25	-2.25	32.50	0.001
Maturity (days)	5A		99.00	97.00	-2.00	23.80	0.001
Number of tillers (m^-2^)	5A		108.20	106.20	-2.00	3.60	0.061
Plant height (cm)	5A		83.60	79.60	-4.00	9.80	0.002
1000 kernel weight (g)	5A		39.00	39.00	0.00	0.20	0.694
Test weight (kg hL^-1^)	5A		76.90	77.20	0.30	4.80	0.030
Grain yield (Mg ha^-1^)	5A		4.77	4.73	-0.04	0.27	0.605
Grain protein content (%)	5A		12.35	12.40	0.05	0.22	0.402

* The difference in leas squares means was calculated by subtracting values for ‘Attila’ type alleles from those of ‘CDC Go’ type alleles. Positive and negative values for grain yield, grain protein content, test weight, kernel weight and number of tillers indicate that the favorable alleles originated from ‘CDC Go’ and ‘Attila’, respectively; for flowering, maturity and plant height, positive and negative values indicate the opposite (the favorable alleles originated from ‘Attila’ and ‘CDC Go’, respectively), because selection is made against higher values (against late flowering, late maturity and taller plants).

## Discussion

### Effects of marker density and management on QTL detection

In one of our previous studies in the ‘Attila’ and ‘CDC Go’ RIL population [9], we genotyped the population with 579 DArT markers and *Rht-B1*, and phenotyped them at three environments grown under conventional management. That study identified a total of three QTLs associated with the averaged phenotypic data over three environments, which included a QTL for grain yield on chromosome 6A, plant height on 4B and test weight on 1A. Each QTL accounted for 10.9 to 19.2% of the phenotypic variance [9] and 33.1 to 45.9% of the genetic variance (Table 2). However, no QTL was identified for the other 5 traits recorded over three environments. Our previous study was based on a total map length of 2045 cM, with an overall average map distance among adjacent markers (inter-marker interval) of 3.5 cM [9], while the present study was based on 1203 informative SNP and gene specific markers, which increased the genome coverage over two fold (3442 cM instead of 2045 cM) and decreased average inter-marker interval by 0.6 cM. In the present study conducted using phenotypic data averaged across seven conventionally managed environments, we found a total of 14 QTLs associated with all agronomic traits (Table 3). Thus, the SNP-based high density markers not only increased the number of detected QTLs from 3 to 14, but also the percentage of phenotypic and genetic variance explained for all traits except plant height and grain yield (Table 2). A similar trend was observed in the ‘Attila’ x ‘CDC Go’ RIL population phenotyped under organic management [20].

We recently reanalyzed the phenotypic data generated across three organically managed environments using the same number of markers as the present study and identified a total of 16 QTLs, of which 13 QTLs were not detected using the DArT-based low marker density [20]. The total phenotypic and genetic variance explained by all QTLs associated with each trait under organic management varied from 9.3 to 39.4% and from 24.6 to 96.8%, respectively [20], which was much greater than our previous study using DArT-based linkage maps [9]. In general, the Wheat 90K SNP array [19] has significantly improved QTL detection both under organic and conventional management systems, because many gaps in previous low-density DArT-based maps were filled by novel SNP markers [20–23]. Given the medium to high heritability for some traits, such as flowering time (0.73) and plant height (0.62), however, a major proportion of the genetic variance for these traits still remained unexplained, which may partly be due to low polymorphism on the D genome (S1 Table). Our results from the present and previous studies [20, 21], together with others [22, 23] clearly suggest that the wheat 90K SNP array is still not yet an optimal genotyping platform for QTL discovery.

Using the averaged phenotypic data across three organically and seven conventionally managed environments, we uncovered a total of 24 QTLs, of which 6 QTLs were common between the two managements, while the remaining 18 QTLs were detected either under conventional (8) or organic (10) environments. The number of QTLs detected for flowering time, maturity, pant height and tillering remained the same irrespective of the management system and number of environments. For the other traits, we found one or two fewer QTLs for test weight, kernel weight and grain yield, and two additional QTLs for grain protein content under conventional than organic management system. Each QTL identified under organic management individually explained from 5.5 to 18.8% of phenotypic variance, which is similar to the 6.1 to 18.4% phenotypic variation obtained under conventional management. Overall, the total phenotypic variance explained by all QTLs associated with each trait under conventional and organic management system differed from 0.4% for flowering time and plant height to 19.7% for grain protein content. The six common QTLs between the conventional and organic management included one for flowering time on chromosome 5A (*QFlt*.*dms-5A*), two for maturity on 4B and 5A (*QMat*.*dms-4B* and *QMat*.*dms-5A*.*2*), one for plant height on 4B (*QPht*.*dms-4B*) and two for kernel weight on 4A and 6A (*QTkw*.*dms-4A* and *QTkw*.*dms-6A*.*1*). The percentage of phenotypic variance explained by *QFlt*.*dms-5A* and *QPht*.*dms-4B* was the same under organic and conventional management. In the conventional management system, both *QMat*.*dms-5A*.*2* and *QTkw*.*dms-6A*.1 showed reduction in phenotypic variance by 3.2–3.7%, while those of *QMat*.*dms-4B* and *QTkw*.*dms-4A* showed an increase by 5.3–10.0% as compared with organic management. In a previous study conducted using the DArT-based low density markers [9], only a single QTL on chromosome 4B (*QHt*.*dms-4B*) associated with plant height was common between the two management systems. Although we identified five more QTLs that were common between the organic and conventional management systems using the SNPs than the DArTs, most QTLs still remained management specific.

### Comparisons of QTLs with other studies

In western Canada, where the growing season is short and days are long, the development of early maturing wheat cultivars is important to avoid frost damage, which can affect both yield and grain quality [31, 32]. In the present study, we found one coincident QTL associated with both flowering (*QFlt*.*dms-5A*) and maturity (*QMat*.*dms-5A*.*2*) between 294 and 298 cM interval on chromosome 5A, which accounted for 14.0–16.8% of the phenotypic variance for both traits, which is equivalent to a reduction in both flowering and maturity time up to 3 days (Table 3). One of the vernalization response genes, *Vrn-A1*, maps on the long arm of chromosome 5A [25, 33] and directly influence both flowering time and maturity [34, 35]. In the present study, the *Vrn-A1* gene is either one of the flanking markers for *QFlt*.*dms-5A* and *QMat*.*dms-5A*.*2* or mapped 1–2 cM proximal to the coincidental QTL region, which could be due to one of the following reasons. The first possibility is that the coincidental QTL may be the same as the *Vrn-A1* gene, which is partly supported by high LD values (0.70 ≤ r^2^ ≤ 0.75) between the *Vrn-A1* gene specific marker and the SNPs that mapped within the QTL confidence interval on chromosome 5A; however, the LD values were not high enough to confidently suggest that the QTL is the same as the *Vrn-A1* gene. In addition, the QTL only accounted for 14–17% of the phenotypic variance, which is not a typical effect for single genes. The alternative scenario is that the *Vrn-A1* gene may be tightly linked with the coincident QTL region, but the statistical methods used for linkage analysis and QTL mapping failed to discriminate them due to small number of recombinants in our RIL mapping population to break up the linkage, which has been discussed in detail in our previous study [20].

One of the QTLs for plant height (*QPht*.*dms-4B*) mapped at 82 cM on chromosome 4B (Table 3). In hexaploid wheat, dwarfing has been achieved mainly through the introduction of *Rht-B1b* on chromosomes 4B and *Rht-D1b* on chromosomes 4D, which have been introduced in many cultivars grown worldwide [26, 36, 37]. In a RIL population derived from ‘Cutler’ and ‘AC Barrie’, our group has recently reported a very consistent major effect QTL adjacent to *Rht-D1b* gene on chromosome 4D that accounted for 38% of the phenotypic variance for plant height across five environments, which was equivalent to a reduction in plant height by 13 cm [21]. In the ‘Attila’ x ‘CDC Go’ RIL population, *QPht*.*dms-4B* accounted for 18% and 10% of the phenotypic variance across combined conventionally and organically managed environments, respectively. In both organic and conventional management conditions, however, *QPht*.*dms-4B* exhibited either strong linkage or pleiotropic effect with a QTL for maturity (*QMat*.*dms-4B)* and grain protein content (*QGpc*.*dms-4B*) (Table 4). This coincidental QTL mapped between 79 and 86 cM on 4B and shortened plant height by 7.6 cm, but increased maturity by two days and decreased grain protein content by 0.3% (Table 4). This QTL mapped adjacent to *Rht-B1* gene in our previous study conducted using the DArT-based low density markers [9] and 33.5 cM proximal to the *Rht-B1* in the present study conducted using the SNP-based high density markers. Pairwise LD values between the *Rht-B1* gene specific marker and all SNP markers that mapped on the QTL region on 4B varied from 0.03 to 0.17 (data not shown), which is too low to suggest any association between the coincidental QTL and the *Rht-B1* gene.

We identified a single QTL from ‘Attila’ for tillering (*QTil*.*dms-6A*.*1*) on chromosome 6A that accounted for 11.2% and 44.9% of the phenotypic and genetic variance, respectively. In spring wheat, QTLs associated with tillering have been reported near *Gli-A2* (Xpsr10) on the short arm of chromosome 6A and several other chromosomes [38]. For grain yield, we found a single QTL on chromosome 2D (*QYld*.*dms-2D*.*2*) that explained 9.3% and 22.1% of the phenotypic and genetic variance across seven environments, which is equivalent to an increase in grain yield by 376 kg ha^-1^ (Table 3). The photoperiod sensitivity gene (*Ppd-D1*) on chromosome 2D has been the focus in breeding for early maturing wheat cultivars to better adapt to their environments [39, 40]. Different studies have also reported QTLs associated with grain yield on chromosome 2D [21, 40, 41]. In another study using a RIL population derived from a cross between ‘Cutler’ and ‘AC Barrie’, our group has recently reported a major coincident QTL associated with flowering time, maturity and grain yield on 2D, flanked by *Ppd-D1* gene, which resulted in a reduction in maturity up to 5 days, but showed a yield penalty of 436 kg ha^-1^ [21]. In the present study, however, the QTL associated with grain yield across the seven environments mapped 66 cM distal to the *Ppd-D1* gene, which is genetically far. Pairwise LD values between the *Ppd-D1* gene and all SNP markers that mapped around *QYld*.*dms-2D*.*2*varied from 0.002 to 0.035 (data not shown), which is too small to suggests any association between *QYld*.*dms-2D*.*2* and the *Ppd-D1* gene.

In our previous study using DArT markers, we reported (i) a single QTL associated with test weight on chromosome 1B (*QTwt*.*dms-1B*) that explained 8.3% of the phenotypic variance across three environments; and (ii) two QTLs associated with kernel weight on chromosome 4A and 6A that together explained 18.7% of the phenotypic variance across three environments [9]. In the present study using SNP-based high density markers and phenotypic data across seven environments, we uncovered (i) two QTLs (*QTwt*.*dms-5A and QTwt*.*dms-5B*.*3*) associated with test weight that individually explained 6.1 and 10.1%, respectively (Table 3) and altogether accounted for 16.2% of the phenotypic variance and 46.4% of the genetic variance (Table 2), and (ii) four QTLs associated with kernel weight (*QTkw*.*dms-4A*, *QTkw*.*dms-6A*.*1*, *QTkw*.*dms-6D*.*2* and *QTkw*.*dms-7B*.*1*) that individually explained 6.7–12.1% of the phenotypic variance (Table 3), and altogether accounted for 35.4% of the phenotypic and 90.8% of the genetic variance (Table 2). Only two of the four QTLs associated with kernel weight both on chromosomes 4A (*QTkw*.*dms-4A*) and 6A (*QTkw*.*dms-6A*.*1*) were common between the present and previous [9] studies. QTLs for test weight have also been reported on several chromosomes, including chromosomes 1A, 1B, 1D, 2D, 3B, 3D, 4A, 4D, 5A, 5D, 6B, and 7A [42–45]. In a RIL population derived from ‘Chuan 35050’ × ‘Shannong 483’, four QTLs have been reported for kernel weight, which includes a consistent QTL on chromosome 6A (*QTkw*.*sdau-6A*) that explained from 6.1 to 13.2% of the phenotypic variance across three environments [46].

The development of wheat cultivars with high grain protein content or high proportion of the essential amino acids have been one of the target traits in wheat breeding [47]. In the present study, we found two QTLs associated with grain protein content on chromosomes 2D (*QGpc*.*dms-2D)* and 4B (*QGpc*.*dms-4B*) that individually explained 13.4% and 6.3% of the phenotypic variance, respectively, and altogether accounted for 18.7% of the phenotypic variance (Table 3) and 71.7% of the genetic variance across seven environments (Table 2). *QGpc*.*dms-2D* mapped 61–65 cM distal to the *Ppd-D1* gene. Neither *QGpc*.*dms-2D* nor *QGpc*.*dms-4B* identified in the present study was reported in our previous studies in the ‘Attila’ x ‘CDC Go’ population evaluated under organic management [9, 20]. As discussed above, the QTL for grain protein content on 4B coincided with maturity and plant height, which could be due to tight linkage or pleiotropic effect. The QTL for grain yield on 2D (*QYld*.*dms-2D*.*2*) mapped 5 cM distal to the QTLs for grain protein content (*QGpc*.*dms-2D*), but the genetic confidence interval between *QYld*.*dms-2D*.*2* (62–70 cM) and *QGpc*.*dms-2D* (59–63 cM) showed an overlap; both QTLs mapped 61–66 cM distal to the *Ppd-D1* gene, so neither *QYld*.*dms-2D*.*2* nor *QGpc*.*dms-2D* are associated with the *Ppd-D1* gene. RILs with ‘Attila’ alleles at the two flanking markers of *QYld*.*dms-2D*.*2* produced 375.7 kg ha^-1^ more grain yield but 0.3% lower grain protein content than those with ‘CDC Go’ alleles (Table 2). QTLs for grain protein content have been previously reported on several chromosomes [48, 49].

## Conclusions

The increase in marker density was highly useful in detecting 14 QTLs of which 13 QTLs were not detected in our previous study using the DArT-based low marker density. However, over 50% of the genetic variance of five traits (flowering time, plant height, test weight, grain yield and number of tillers) still remained unexplained even after doubling marker density, which may be due to gaps on some chromosomes and/or lack of SNP polymorphism on some chromosomes, such as 3D and 4D. Only 6 of the 14 QTLs identified under conventional management were common with those identified under organic management, which clearly suggest the management specificity of the majority of QTLs. Our results from the different studies conducted in the ‘Attila’ × ‘CD Go’ population using both low and high density markers suggest the need in conducting management specific gene discovery studies and provides insight on difficulty in directly utilizing genes or major effect QTLs identified based on phenotypic data from conventional management to make selection under organic management.

## Supporting information

S1 Fig Frequency distribution of least square means of 167 recombinant inbred lines (RILs) and parents evaluated for 8 agronomic traits averaged across seven environments under conventional management system. The arrows indicate values of the two parents. See Table 1 for details.
(DOCX)

S1 Table Summary of the 5,667 SNPs and three gene specific markers (*Ppd-D1*, *Rht-B1* and *Vrn-A1*) integrated in to the linkage map of ‘Attila’ ×‘CDC Go’ RIL population and a subset of 1203 markers selected for QTL mapping [20]. (XLSX)

S2 Table Summary of the 16 QTLs associated with seven agronomic traits evaluated across three combined environments (2008–2010) under organic management system [20]. (DOCX)
