# Supplementary material for: QTLs associated with agronomic traits in the Attila × CDC Go spring wheat population evaluated under conventional management
Source: PLoS One. 2017 Feb 3;12(2):e0171528. doi: 10.1371/journal.pone.0171528 (PMC5291526; doi:10.1371/journal.pone.0171528)
Supplement: S1 Fig — The arrows indicate values of the two parents. See Table 1 for details. (DOCX) [file pone.0171528.s001.docx]

**S1 Figure**. Frequency distribution of least squares means of 167 RILs evaluated for 8 traits across seven environments (2008-2014) under conventional management system. The arrows indicate values of the parents: CDC Go (C) and Attila (A). See Table 1 for details.

A

C

C

A

A

C

A

C

C

A

C

A

C

A

C

A
