# Supplementary material for: QTLs associated with agronomic traits in the Attila × CDC Go spring wheat population evaluated under conventional management
Source: PLoS One. 2017 Feb 3;12(2):e0171528. doi: 10.1371/journal.pone.0171528 (PMC5291526; doi:10.1371/journal.pone.0171528)
Supplement: S2 Table — (DOCX) [file pone.0171528.s003.docx]

**S2 Table.** Summary of 16 QTLs associated with seven agronomic traits based on 167 recombinant inbred lines evaluated across three environments (2008-2010) under organic management system [18].

| Trait | QTL | Chrom | Position (cM) | Confidence interval (cM) | Left marker | Right marker | LOD | R^2^ (%) | Additive effect* | Phenotypic difference** |
| --- | --- | --- | --- | --- | --- | --- | --- | --- | --- | --- |
| Flowering | *QFlt.dms-5A.2* | 5A | 297 | 293.5-297.5 | Kukri_c12384_430 | wsnp_Ex_c22727_31934296 | 6.8 | 17.2 | -1.1 | -2.2 |
| Maturity | *QMat.dms-4B* | 4B | 79 | 77.5-79.5 | BobWhite_c5694_1201 | wsnp_Ra_c3790_6990678 | 2.6 | 5.9 | 0.9 | 2 |
| Maturity | *QMat.dms-5A* | 5A | 298 | 297.5-299.5 | wsnp_Ex_c22727_31934296 | wsnp_Ex_rep_c66689_65010988 | 7 | 17.2 | -1.4 | -2.7 |
| Plant height | *QPht.dms-4B* | 4B | 81 | 79.5-80.5 | RAC875_c3790_429 | Tdurum_contig29054_113 | 7.5 | 18.8 | -3.7 | -7.2 |
| No. of tillers | *QTil.dms-4A* | 4A | 0 | 0-1.5 | Excalibur_c82040_91 | wsnp_Ra_rep_c70233_67968353 | 3.5 | 9.3 | 2.7 | 5.3 |
| Grain yield | *QYld.dms-5B* | 5B | 37 | 36.5-38.5 | TA002682-0717 | BobWhite_c26082_80 | 3.2 | 7.5 | -153.5 | -287.3 |
| Grain yield | *QYld.dms-7A* | 7A | 25 | 23.5-27.5 | BobWhite_c41815_145 | wsnp_Ex_c40247_47349166 | 2.5 | 5.8 | -135.3 | -281 |
| Test weight | *QTwt.dms-1A* | 1A | 81 | 79.5-81.5 | Kukri_c82555_88 | wsnp_Ex_c31983_40709607 | 3.2 | 7.5 | -0.3 | -0.6 |
| Test weight | *QTwt.dms-3A* | 3A | 168 | 156.5-181.5 | BobWhite_c13293_107 | BS00022452_51 | 2.6 | 10.3 | -0.4 | -0.8 |
| Test weight | *QTwt.dms-4A* | 4A | 15 | 12.5-16.5 | JD_c6741_185 | Excalibur_c76268_300 | 2.8 | 6.5 | -0.3 | -0.6 |
| Test weight | *QTwt.dms-5B* | 5B | 204 | 201.5-205.5 | Kukri_c43972_367 | Kukri_c46932_65 | 3.7 | 8.6 | -0.3 | -0.6 |
| Kernel weight | *QTkw.dms-1B* | 1B | 16 | 14.5-17.5 | Ku_c1932_1583 | Excalibur_rep_c103592_565 | 3.1 | 5.5 | -0.7 | -1.3 |
| Kernel weight | *QTkw.dms-4A* | 4A | 120 | 118.5-120.5 | wsnp_Ex_c7899_13416443 | wsnp_Ex_rep_c97236_84366722 | 3.8 | 6.8 | 0.7 | 1.8 |
| Kernel weight | *QTkw.dms-6A* | 6A | 79 | 77.5-80.5 | wsnp_Ku_rep_c112734_95776957 | BS00036878_51 | 6.5 | 12.2 | 1 | 2.1 |
| Kernel weight | *QTkw.dms-6B.1* | 6B | 6 | 4.5-12.5 | wsnp_Ex_c56091_58346859 | wsnp_JD_c23373_19987039 | 4.7 | 8.5 | -0.8 | -1 |
| Kernel weight | *QTkw.dms-6B.2* | 6B | 37 | 29.5-42.5 | Excalibur_c35713_106 | RAC875_c6837_468 | 3.6 | 6.4 | 0.7 | 1.5 |

* Additive effect is half the difference between the genotypic values of ‘Attila’ minus ‘CDC Go’. **Difference in phenotypic performance of all RILs that had the ‘CDC Go’ alleles at both flanking markers of every QTL to those that had the ‘Attila’ alleles. The difference was calculated only for the three environments combined phenotypic data and the units are number of days for flowering and maturity, cm for plant height, kg ha^-1^ for grain yield, g for test weight, kg hL^-1^ for thousand kernel weight, % for protein content, and number for tillering. Positive and negative additive effect and differences for grain yield, grain protein content, test weight, kernel weight and tillering indicate that the favorable alleles originated from CDC Go and Attila, respectively; for flowering, maturity and plant height, positive and negative values indicate that favorable alleles originated from Attila and CDC Go, respectively, because selection is made against late flowering, late maturity and taller plants.
